# Odontological Society of Great Britain

**Published:** 1886-03

**Authors:** 


ARTICLE III.

### ODONTOLOGICAL SOCIETY OF GREAT BRITAIN

The Annual General Meeting of this Society was held on January 11th, Mr. C. Spence Bate, F. R. S., President, in the chair.

Mr. S. J. Hutchinson related the following case:—In October last a gentleman came to him suffering from almost complete closure of the jaws; he could only separate them about a quarter of an inch, and was, of course, unable to eat. He had been in this condition about a month. Mr. Hutchinson found that the inability to move the jaws was due to muscular contraction, with infiltration and induration of the surrounding tissues, and diagnosed an impacted wisdom tooth, but was unable to make a satisfactory examination of the mouth.

At the patient's next visit gas was administered and the jaws separated to some extent by a screw gag. It was then found that the left lower wisdom tooth was completely buried under the ascending ramus of the jaw; it was also decayed and had an abscess at the root. In order to get at it Mr. Hutchinson felt obliged to extract the adjacent second molar, and this was at once done. After an interval of a few days gas was again administered and an attempt made to dislodge the wisdom tooth, but the patient recovered himself before this could be affected.

Perceiving that the operation would be a difficult one, Mr. Hutchinson arranged with the patient that he should have chloroform at his own house, and this time the tooth was successfully extracted, though not without a good deal of trouble, for the tooth was lying horizontally and buried under the ramus of the jaw.

Mr. Hutchinson thought that the plan of administering chloroform at the patient's own house, instead of at that of the practitioner, reduced the risks and inconveniences of this agent to a minimum. The patient could be operated on undressed and in a recumbent position; he was not fatigued, as he might be when he had to come some distance in a fasting condition, and he was less flurried. Finally, he could lie quietly after the operation and recover himself at his leisure.

Another point of interest in the case was the length of time the contraction persisted after extraction of the tooth. Three weeks after the operation the patient could only open his mouth three-quarters of an inch, and he had, in fact, only just regained the free use of his jaw, though the operation took place in October.

Mr. Charters White said that about two years ago he had been called upon to treat a precisely similar case. Thinking from the amount of indurated swelling, &c., that there must be some disease of the jaw, he sent the patient to Mr. Christopher Heath, but that gentleman sent him back again, saying he considered it a case for a dentist, and on examining the patient more carefully Mr. White discovered that the cause of the mischief was an impacted wisdom tooth, embedded in the ascending ramus of the lower jaw. The patient could not separate his teeth more than a quarter of an inch, so that it was difficult to introduce forceps, and still more so to manipulate them in the mouth, but Mr. White managed to reach the tooth with some long narrow curved forceps, and to raise it out of its socket, and at the patient's next visit, the swelling having somewhat diminished, he succeeded with the same forceps in removing it altogether.

Mr. Henri Weiss said that in five or six cases of prolonged operations in the mouth he had used the well-known A. C. E. mixture, composed of alcohol one part, chloroform two parts, and ether three parts, by measure, and had found it to act very satisfactorily. The patients recovered quickly and there were no bad after-effects.

Mr. R. H. Woodhouse said he knew that the extraction of lower wisdom teeth was sometimes attended with a good deal of difficulty, and he believed that this arose from overlooking the fact that these teeth, when misplaced, were almost invariably inclined to the inner side of the ramus. He found that by inserting an elevator on the outer side, and making a continuous movement inwards, he could dislodge them without any trouble. He believed the difficulty arose from making an outward movement; it should be entirely inwards.

Mr. A. S. Underwood said that, in the years 1878 and 1879, Dr. Bodecker, of New York, published in the *Dental*

*Cosmos* some papers on the microscopical anatomy of the teeth, in which, amongst other things, he asserted that the presence of protoplasm between the fibres of the enamel could be demonstrated by staining sections with chloride of gold. He described his process as follows :—He first decalcified the teeth by means of chromic acid, then cut sections, and stained these by placing them in a solution of chloride of gold, and exposing them to sun-light for twenty-four hours or more. Now up to that time it had always been stated that chloride of gold would only stain tissues which were absolutely fresh. The text-books said it was useless to attempt to stain tissues which had been deprived of life for more than an hour, and a distinguished microscopist to whom Mr. Underwood applied for information on the subject replied that it was hopeless to attempt staining with the chloride unless the tissues were fresh, and even in that case four out of five of his sections would turn out failures.

Wishing to verify Dr. Bodecker's observations, if possible, Mr. Underwood undertook a series of experiments in order to ascertain whether it was possible to stain decalcified sections with this reagent, and what was the best method of using it.

His results with the method described by Dr. Bodecker had been uniformly unsatisfactory; he could not get a single section which showed anything clearly. But he found, nevertheless, that any section could be stained, and that it really did not matter whether it was fresh or not. The method which he had found the best, and which he had finally adopted, was as follows:

He immersed the section, whether cut from a decalcified tooth or ground down from a hard one, in a solution of carbonate of soda for an hour. Then he placed it in a solution of chloride of gold, which must be neutral, and left it in the dark for another hour. It was then again placed in the carbonate of soda solution for a few minutes, and then transferred to a one per cent. solution of formac acid, and kept warm over a water bath for about an hour and a half. Finally the section was mounted in glycerine jelly, not in Canada balsam. Sections which have been decalcified by chromic acid took longer to stain than those which were fresh, but the whole process only occupied from three to four hours, instead of at least twenty-four as in the old method, and the result would be found far more satisfactory. The usual needles, or any steel instrument, must not be used for manipulating the sections; some non-metallic substance, such as a quill tooth-pick, should be used instead.

He found that the most satisfactory method of grinding down hard sections was to grind them tolerably thin against a fine corundum wheel, and afterwards to finish with an Arkansas wheel. In this way the section could be ground down to any required thinness with little risk of injury.

Mr. Charters White said he gathered from Mr. Underwood's remarks, that though it was possible to stain a decalcified section, it was better to grind down a fresh section and stain at once.

The President remarked, that grinding down a hard section between two Arkansas stones saved both the operator's time and his finger.

Mr. Underwood replied that fresh sections were not only easier to stain, but were in all respects much more satisfactory than those which had been decalcified, since the former presented the tissues in their natural condition, whilst in the latter they were more or less affected by time and by the reagents. Consequently, when decalcified specimens were used, there was always a doubt whether the appearances seen were really natural, or whether they were the effect of the reagents. He thought the use of the finger was the safer way of finishing a thin section, and if necessary the finger could be protected by attaching the section to a piece of cork or rubber.

Dr. George Field drew attention to some samples of Dennison's absorbent cotton, an American preparation. He had given it a thorough trial, and was convinced that there was nothing which surpassed it as an absorbent for dental purposes.

He wished to suggest a new use for cocaine—new, at least, to some of those present—viz., in the fixing of the rubber down by means of a ligature around the tooth, especially in the cases frequently met with where it was necessary to force both the rubber and ligature between the tooth or teeth and gum, on the approximal and buccal surfaces of the former. His method of using it was as follows: He first thoroughly dried the cavities and the adjacent gum margins, then by means of a wedge-shaped piece of wood he applied the cocaine between the teeth and the gums, first adjusting a napkin as a protection from moisture. He preferred to use the crystals. Then he prepared the rubber, elastic bands, weights, ligatures, &c., and when everything was ready to hand he made another application of the cocaine. On now proceeding to adjust the dam, it would be found that the ligatures could be forced well under the gum with but little, if any, pain to the patient, provided that the application had been properly made. This operation, which, though absolutely essential for the success of fillings in the position named, was usually exceedingly painful, was thus rendered almost painless; a good view of the margins of the cavity was thus obtained, with dryness, and it greatly facilitated the removal of all surplus material, overhanging the margins of the cavity, an oversight which, in his experience, was the cause of more failures than any other defect in filling operations.

He also offered a few general remarks on the question of the extraction or the retention of roots. When, he asked, should roots be extracted, when retained? When was it advisable to pivot, and when not? Preparatory to the insertion of an artificial denture all roots which could not be put into a good healthy state, fit to receive a crown, should be extracted. In the case of patients who, it might reasonably be expected, would not take sufficient care to keep their teeth, roots, and gums in a cleanly condition, it was wiser to extract all roots; otherwise in from six to twelve months the result of the want of judgment would be seen in swollen face and gums, abscesses, &c., and a state of mouth generally which was a source of great discomfort to the patient himself, disgusting and offensive to his friends, and discreditable to the operator.

He expected to meet with the usual objection—the consequent absorption of the alveoli, &c. But this should have no weight in comparison with the inevitable bad results just named; in addition to which there could be little doubt that the quantity of suppurative matter constantly passing into the stomach must be prejudical to the health of many patients. If the roots of any of the ten anterior teeth of the upper jaw were strong, pivoting in the best possible manner should be given the preference over a plate, as being less liable to injure other sound teeth and of greater practical service, provided the operation be performed with even a moderate degree of skill; giving special attention to the stopping of the foramen of the root, obtaining a good joint between the root and crown, having no shoulder either of root or crown, and lastly removing every particle of the cement used for fixing the crown which may have been pressed out at the joint.

Speaking from his own observation, he had never yet seen a case for the insertion of a full denture, the conditions or circumstances of which would warrant the retention of the roots; whereas he had met with cases in which the retention of numerous diseased roots covered by a plate had proved prejudicial to the general health of the patient.

He did not present these suggestions as being anything new, but rather to call attention to the fact, which at times seemed to be lost sight of, that the mouth should be treated in the same way as any other part of the body, and that it was the duty of the dental practitioner to maintain it in a healthy condition by every means in his power, therapeutic as well as mechanical.

Mr. Storer Bennett showed a lower jaw, found at Bath some years ago amongst Roman remains, which had been presented to the Museum by Mr. Forsyth. On comparing it with a typical modern specimen several differences would be apparent, especially the distances between the condyles and the large size of the ascending rami. There was but slight mental development, and the teeth were not quite regular; they were much worn, but there were no signs of caries, and, contrary to what might have been expected, the wisdom teeth were small.

Dr. George Cunningham showed some specimens illustrating the difficulties and disappointments of Continuous Gum Work. He had used the same furnace (Verrier's) throughout, the details of the process had been carried out in the same way and with the same amount of care, and yet after a period of success, when he thought he had conquered all difficulties, several cases in succession had turned out badly, the enamel being unequally fused and cracked on the outside of the plate. He could not himself explain the cause of his non-success, nor could he get any one else to explain it. The only difference between the good plates and the bad was in the enamel used, though both had been obtained from the same makers, the S. S. White Company, but that used for the successful cases had been on hand a long time, whilst new enamel had been used for the failures. He should be very glad if any one present could tell him whether their experience had been at all similar, or could enlighten him as to the probable cause of his failures.

He wished also to call the attention of the Society to Dr. Land's suction chamber; he handed around a denture made according to his pattern. Dr. Land's suction chamber was large but shallow. He himself had for some time past altogether abandoned the use of these chambers and used the Fulsome ridge; but lately he had been induced to make some comparisons between the two, and had found Dr. Land's method of great use, and he could therefore recommend others to give it a trial.

Dr. Walker said he had met with the same difficulty in firing continuous gum cases, using Verrier's muffles. He would suggest that the bad results were due to the unequal temperature of different parts of the furnace, and that this might be obviated by having a better supply of gas. He thought that if Dr. Cunningham would have a larger supply pipe fitted, not less than ¾ inch diameter, inside measurement, the heat would be equalized, and he would meet with no more failures of this kind.

Mr. D. Hepburn called attention to the following plan for improving the adhesion of suction plates. All must have experienced the difficulty which was not unfrequently met with in establishing the confidence of patients in suction plates, especially when first applied. Even with the most perfect model, the most accurately adjusted arrangement will often at the first offset show no tendency whatever to adhere to the gum, and the patient may have to undergo many days of discomfort before adhesion is established. He had tried to overcome this difficulty by coating plates with various substances of an adhesive nature, in order to spare the patient a disagreeable ordeal, and he was aware that similar attempts had been made by many other practitioners. Thus he had tried sprinkling the plate with flour and painting it with various gums and mucilages, but with little success, most of these substances being rapidly dissolved and washed away. For about a year, however, he had employed powdered gum tragacanth with the most satisfactory results. Indeed, the most refractory plates, when this substance was used, would adhere with a certain amount of tenacity, and frequently could not be dislodged without a considerable effort.

The best method of application was to keep the powder in a bottle with a piece of muslin tied over the mouth, and to sprinkle the plate with a thick layer of the powder before putting it into the mouth. The saliva would in a short time convert the tragacanth into a glutinous and almost tasteless layer which would remain for days. In obstinate cases the patients could themselves apply the powder daily, and found much comfort from so doing.

This use of tragacanth had been suggested to him by a patient of great ingenuity, and he had never met with any substance which would act so efficaciously. Having experienced its utility himself, he wished to suggest its employment, for the purpose referred to, to other members of the Society.

Mr. R. H. Woodhouse said he had found powdered gum arabic of some use in such cases, but at the suggestion of Mr. Hepburn he had lately used the powdered gum tragacanth and had found this very much better.—*Dental Record.*

